# Reconstruction of a High-Grade Osteosarcoma in the Proximal Tibia With Transphyseal Resection Using an Extracorporeal Irradiation and Vascularized Fibula Autograft: A Case Report

**DOI:** 10.7759/cureus.87651

**Published:** 2025-07-10

**Authors:** Arın Celayir, Muhhammed R Hosbas, Mahmut K Ozsahin, Huseyin Botanlioglu

**Affiliations:** 1 Department of Orthopedics and Traumatology, Cerrahpasa Faculty of Medicine, Istanbul University-Cerrahpasa, Istanbul, TUR

**Keywords:** extracorporeal irradiation, high-grade osteosarcoma, limb-salvage surgery, reconstructive orthopedic oncology, vascularized fibula graft

## Abstract

This case report presents the treatment of a 13-year-old male patient diagnosed with high-grade osteosarcoma of the proximal tibia. Management involved a combination of transphyseal resection, extracorporeal irradiation, and reconstruction using a vascularized fibula autograft. The tumor was excised with clear margins, as confirmed intraoperatively through frozen section analysis. The resected tibial segment was sterilized using 50 Gy extracorporeal irradiation and subsequently reimplanted. To restore structural integrity and vascularization, a 14 cm pedicled fibula graft was harvested and inserted intramedullary, then stabilized with plates and screws. This multidisciplinary approach, involving orthopedic oncology and microsurgical reconstruction, demonstrates an innovative limb-salvage strategy that preserves function while minimizing the risk of recurrence. The case highlights the feasibility and potential advantages of combining extracorporeal irradiation with vascularized bone grafting in the management of complex osteosarcomas, particularly in skeletally immature patients.

## Introduction

High-grade osteosarcoma is the most common primary malignant bone tumor, accounting for approximately 20% of all primary bone cancers [1]. It typically arises in the metaphyseal regions of long bones, with the distal femur, proximal tibia, and proximal humerus being the most frequently affected sites [2]. This tumor predominantly affects adolescents and young adults during periods of rapid skeletal growth, although it can occur at any age. High-grade osteosarcoma is characterized by aggressive behavior, rapid growth, and a strong propensity for early hematogenous metastasis, most commonly to the lungs [3].

The standard of care involves a multidisciplinary approach, including neoadjuvant chemotherapy to reduce tumor burden, wide surgical resection for local control, and adjuvant chemotherapy to eliminate microscopic residual disease [4]. Recent advances in limb-salvage techniques have improved functional outcomes without compromising oncological safety. Approaches such as tumor resection followed by extracorporeal irradiation and reconstruction with vascularized grafts offer promising options for complex cases. Despite these advancements, high-grade osteosarcoma remains a significant clinical challenge due to its high recurrence rate and demanding management requirements, underscoring the need for comprehensive care to optimize both survival and quality of life.

## Case presentation

**Figure 1:**
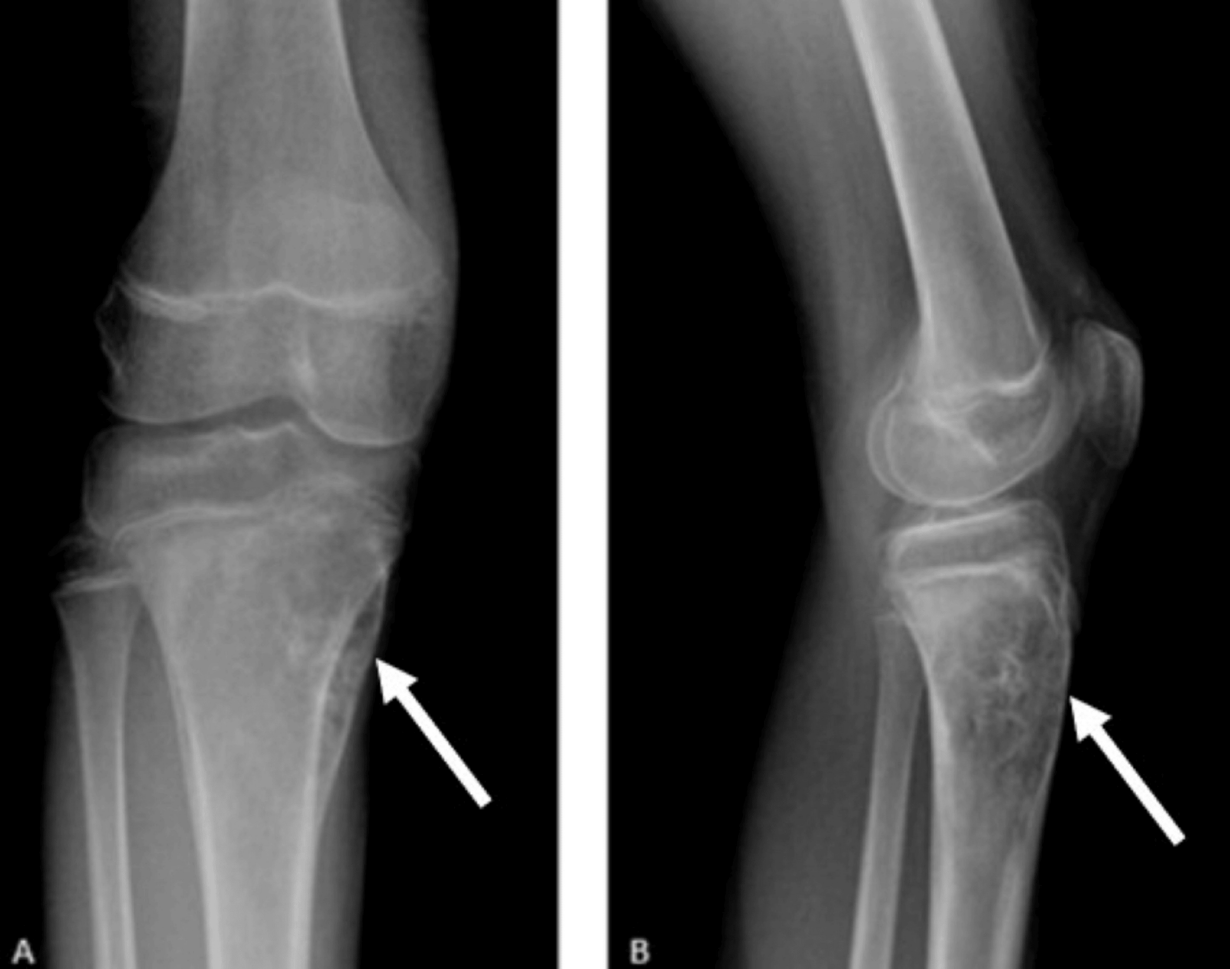
Preoperative radiographs obtained at the time of the patient’s initial presentation. (A) Anteroposterior view demonstrating a lytic-sclerotic lesion in the proximal tibia. (B) Lateral view showing the same lesion with cortical disruption and periosteal reaction. White arrows indicate the lesion

**Figure 2:**
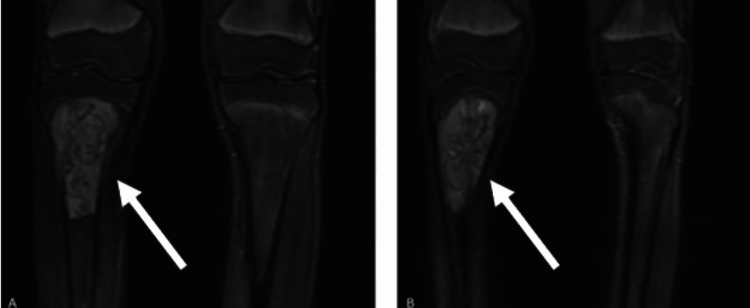
Coronal MRI images of the right knee. (A,B) A heterogeneous mass is observed in the proximal tibia, demonstrating cortical destruction anteriorly with extension into the surrounding extraosseous soft tissues. White arrows indicate the lesion MRI: magnetic resonance imaging

**Figure 3:**
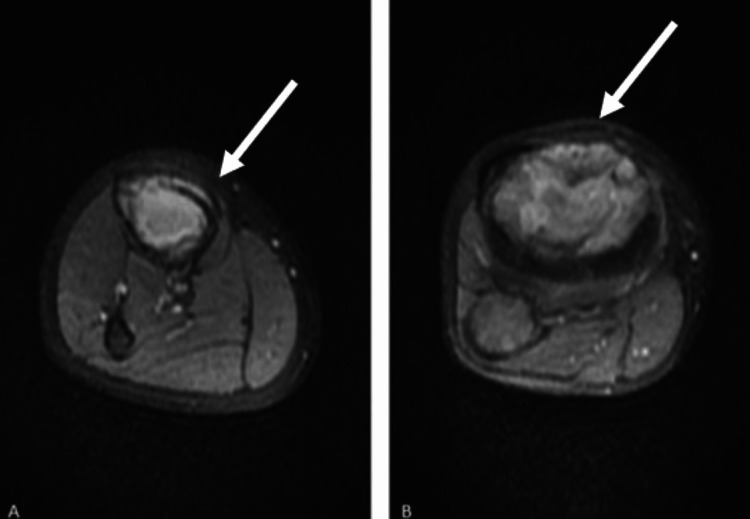
Axial MRI images of the right knee. (A) A soft tissue mass is seen in the proximal tibia with evidence of anterior cortical destruction. (B) The lesion extends into the surrounding extraosseous soft tissues. White arrows indicate the lesion MRI: magnetic resonance imaging

A 13-year-old male patient presented to our clinic with complaints of right knee pain. His medical history was notable for an ectopic kidney, with no other known comorbidities, and he was not receiving any regular medications. An open biopsy of the right knee had been performed at an external center, yielding a diagnosis of high-grade osteosarcoma. Upon presentation to our clinic, informed consent was obtained from the patient and his family. Current plain radiographs were obtained (Figure 1), and magnetic resonance imaging (MRI) was repeated for further evaluation (Figures 2, 3). In addition, a whole-body positron-emission tomography scan was performed to assess for distant metastases, which revealed no evidence of metastatic disease.

**Figure 4:**
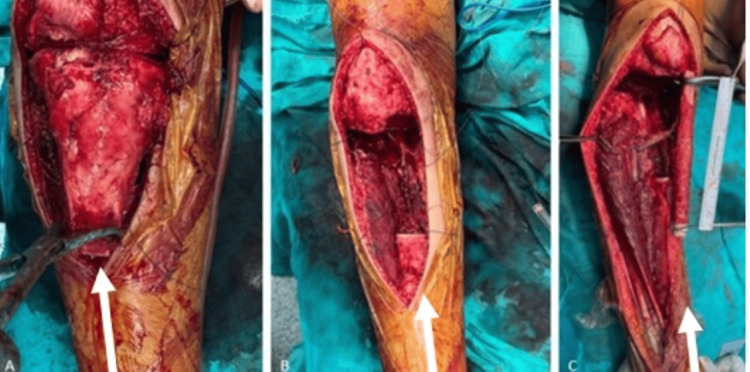
Intraoperative images during proximal tibial resection and fibula graft preparation. (A) Exposure of the proximal tibial lesion prior to resection. (B) Surgical site following en bloc resection of the tumor. (C) Harvested and prepared vascularized fibula autograft prior to reconstruction. White arrows indicate the lesion

MRI evaluation revealed a lesion measuring 92 × 51 × 37 mm located in the proximal metaphysis of the right tibia. The mass extended anteriorly into the extraosseous space through cortical destruction and demonstrated heterogeneous contrast enhancement. It did not invade the joint space and remained at a safe distance from the neurovascular bundle. The case was reviewed at our multidisciplinary tumor board, and it was decided to proceed with wide resection, extracorporeal irradiation, and reconstruction using a vascularized fibula graft. Surgical preparation was completed under sterile conditions, and prophylactic intravenous cefazolin (1.5 g) was administered. A 13-year-old male patient with high-grade osteosarcoma of the proximal tibia underwent wide resection followed by extracorporeal irradiation and reconstruction using a vascularized fibula graft. A 10 cm longitudinal incision was made, and the previous biopsy tract was excised en bloc. The proximal tibial segment was isolated for extracorporeal irradiation, and intraoperative frozen section analysis confirmed negative surgical margins (Figure 4).

**Figure 5:**
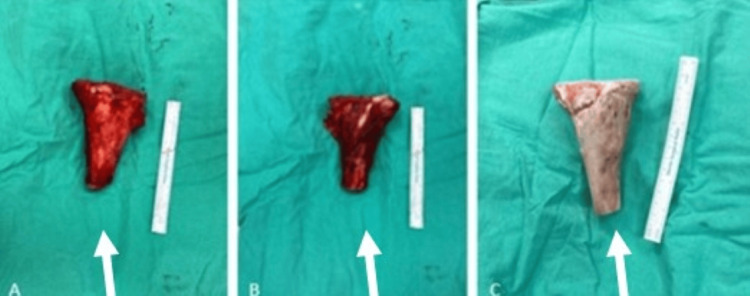
Images of the resected tibial segment before and after extracorporeal irradiation. (A,B) Views of the tibial segment immediately following resection and prior to irradiation. (C) The tibial segment after completion of extracorporeal irradiation and surgical preparation for reimplantation. White arrows indicate the lesion

The excised tibial segment was irradiated with 50 Gy and returned sterile. A 14 cm pedicled fibula graft was harvested, shaped, and placed intramedullary in the tibia (Figure 5).

**Figure 6:**
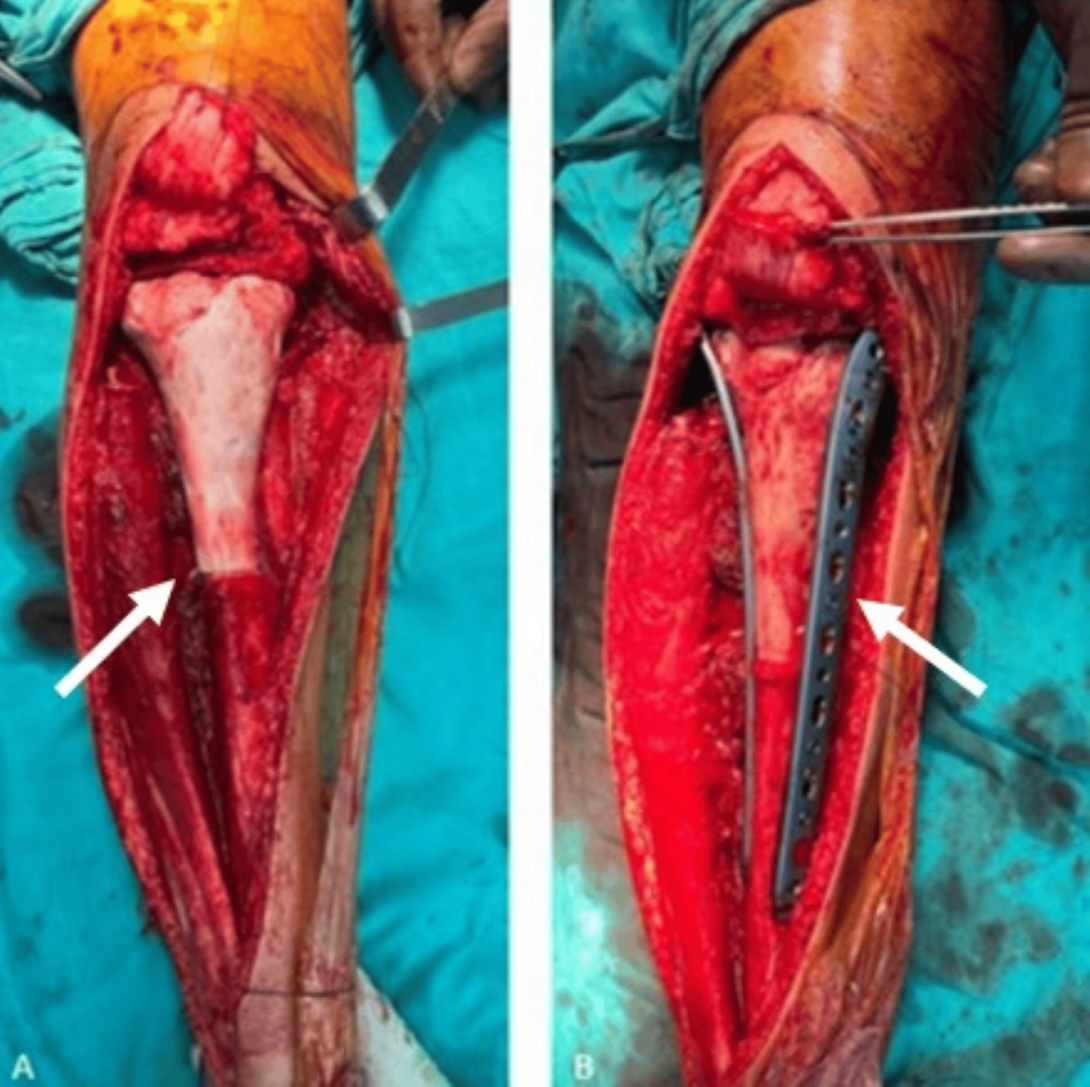
Intraoperative images following reconstruction using a vascularized fibula autograft. (A) The reimplanted tibial segment with the intramedullary fibula graft positioned in place. (B) Final fixation achieved using a plate and screws

Fixation was achieved using plates, screws, and sutures (Figure 6).

**Figure 7:**
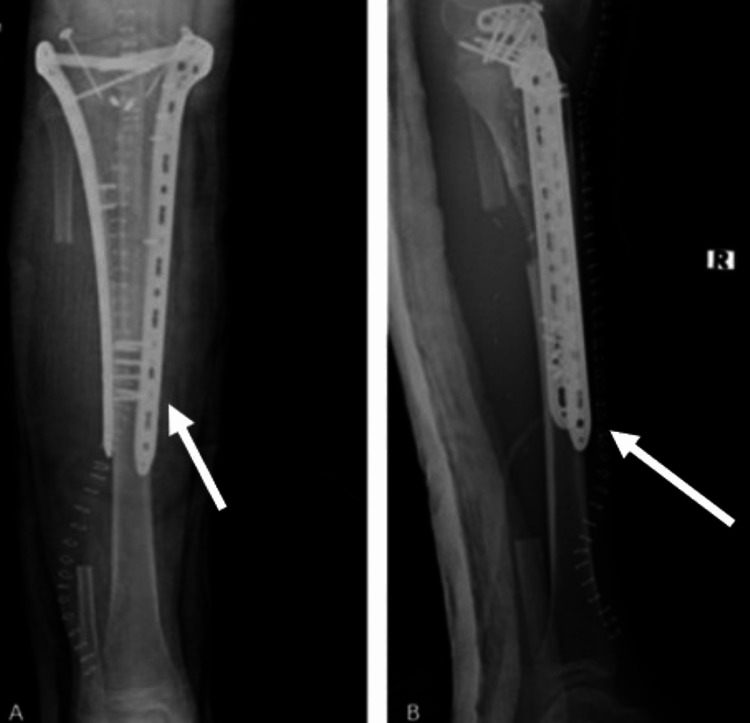
Postoperative images of the patient. (A) AP radiograph showing the appearance after fixation with plate and screws. (B) Lateral radiograph showing the appearance after fixation with plate and screws AP: anteroposterior

Final X-rays confirmed proper reconstruction and implant placement (Figure 7).

**Figure 8:**
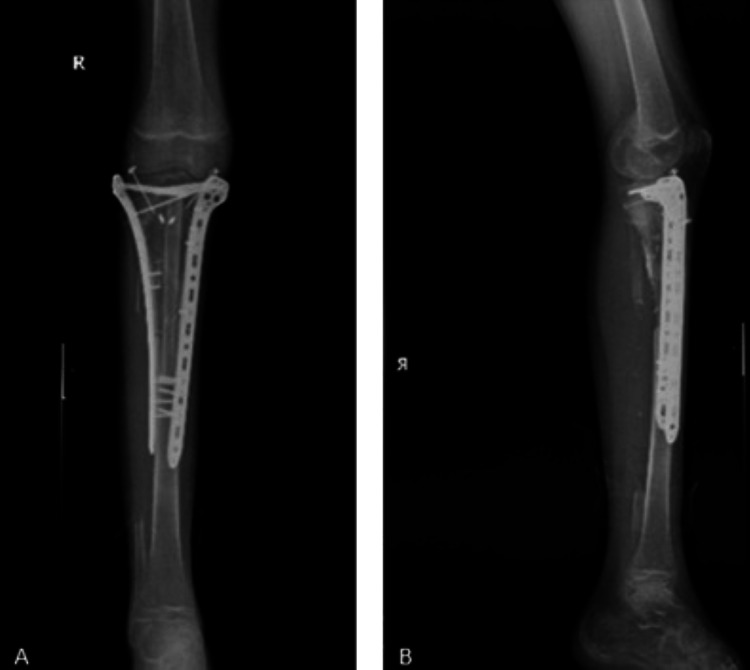
Postoperative images of the patient at the 12th month. (A) AP radiograph view. (B) Lateral view AP: anteroposterior

Postoperative antibiotics were administered. The patient was monitored postoperatively with a long leg splint for four weeks. Weight-bearing was prohibited for approximately six weeks. In the third postoperative week, the patient was referred to medical oncology. The patient received chemotherapy treatment in the postoperative period. At the 12-month follow-up, no distant metastasis or recurrence was detected. No nonunion or fractures were detected in the patient's 12-month follow-up radiograph (Figure 8).

## Discussion

This case illustrates a successful and innovative approach to treating high-grade osteosarcoma of the proximal tibia, emphasizing the advantages of limb-salvage surgery using wide resection, extracorporeal irradiation, and reconstruction with a vascularized fibula autograft [5]. High-grade osteosarcoma poses significant therapeutic challenges due to its aggressive behavior, high risk of local recurrence, and frequent pulmonary metastasis. Optimal management requires a multidisciplinary strategy that integrates surgery, chemotherapy, and, in select cases, radiotherapy.

Historically, amputation was the primary surgical option to ensure complete tumor excision and minimize recurrence risk. Although effective from an oncological standpoint, amputation substantially compromises functional outcomes and quality of life. Advances in surgical techniques have led to a shift toward limb-salvage procedures in appropriately selected patients [6]. Wide resection with negative margins, as achieved in this case, remains essential for ensuring local disease control while preserving limb function.

Reconstructive strategies following wide resection include endoprosthetic replacements, allografts, and biological methods such as autografts. In this case, extracorporeal irradiation enabled sterilization and reimplantation of the patient’s own bone, reducing complications associated with allografts, such as infection, immune rejection, or limited availability. Incorporating a vascularized fibula autograft provided additional biological and mechanical support, promoting long-term graft incorporation and structural integrity.

Systemic chemotherapy plays a pivotal role in managing high-grade osteosarcoma. Neoadjuvant chemotherapy facilitates tumor shrinkage and addresses micrometastatic disease, improving surgical feasibility and outcomes. Postoperative adjuvant chemotherapy is critical for reducing recurrence risk and improving survival. In this case, the patient was referred for medical oncology follow-up to ensure comprehensive systemic treatment [7].

Although osteosarcoma is relatively radioresistant, radiotherapy may be beneficial in selected scenarios, particularly when surgical margins are close or positive [7]. Here, extracorporeal irradiation served as a novel application, sterilizing the tumor-bearing bone ex vivo and preserving its structural characteristics, thus avoiding the drawbacks of in vivo radiotherapy.

Alternative treatments, such as rotationplasty or expandable prostheses, were considered but deemed less suitable due to the patient’s age, tumor location, and the availability of advanced biological reconstruction techniques. Each approach carries unique risks and benefits, highlighting the need for individualized treatment planning.

This case underscores the importance of multidisciplinary care in the management of complex osteosarcomas. The integration of advanced surgical techniques, systemic chemotherapy, and innovative radiotherapeutic applications allowed for effective oncologic control and functional preservation. This approach provides a valuable treatment model for similar challenging cases in pediatric and adolescent populations.

## Conclusions

The treatment of high-grade osteosarcoma requires a careful balance between achieving oncological control and preserving limb function. This case demonstrates that wide resection, extracorporeal irradiation, and reconstruction with a vascularized fibula graft can serve as a successful limb-salvage strategy. While amputation remains a viable option in certain cases, advancements in surgical techniques and the use of systemic chemotherapy have significantly improved functional and oncological outcomes.
